# Dissociating Landmark Stability from Orienting Value Using Functional Magnetic Resonance Imaging

**DOI:** 10.1162/jocn_a_01231

**Published:** 2018-05-01

**Authors:** Stephen D. Auger, Eleanor A. Maguire

**Affiliations:** University College London

## Abstract

Retrosplenial cortex (RSC) plays a role in using environmental landmarks to help orientate oneself in space. It has also been consistently implicated in processing landmarks that remain fixed in a permanent location. However, it is not clear whether the RSC represents the permanent landmarks themselves or instead the orienting relevance of these landmarks. In previous functional magnetic resonance imaging (fMRI) studies, these features have been conflated—stable landmarks were always useful for orienting. Here, we dissociated these two key landmark attributes to investigate which one best reflects the function of the RSC. Before scanning, participants learned the features of novel landmarks about which they had no prior knowledge. During fMRI scanning, we found that the RSC was more engaged when people viewed permanent compared with transient landmarks and was not responsive to the orienting relevance of landmarks. Activity in RSC was also related to the amount of landmark permanence information a person had acquired and, as knowledge increased, the more the RSC drove responses in the anterior thalamus while viewing permanent landmarks. In contrast, the angular gyrus and the hippocampus were engaged by the orienting relevance of landmarks, but not their permanence, with the hippocampus also sensitive to the distance between relevant landmarks and target locations. We conclude that the coding of permanent landmarks in RSC may drive processing in regions like anterior thalamus, with possible implications for the efficacy of functions such as navigation.

## INTRODUCTION

Landmarks are an essential component of our spatial representations of the environment (Burnett, Smith, & May, 2001; Siegel & White, 1975; Lynch, 1960). In recent years there has been increased interest in characterizing the features of landmarks, in particular seeking to identify those traits that are helpful for building environmental representations and that facilitate effective wayfinding (Auger, Zeidman, & Maguire, 2017; Marchette, Vass, Ryan, & Epstein, 2015; Auger & Maguire, 2013; Auger, Mullally, & Maguire, 2012; Konkle & Oliva, 2012; Lew, 2011; Yoder, Clark, & Taube, 2011; Galati, Pelle, Berthoz, & Committeri, 2010; Committeri et al., 2004; Janzen & van Turennout, 2004). The brain areas that process these landmark features have also begun to be scrutinized with a view to understanding the neural evolution of environmental representations and the mechanisms involved (Alexander & Nitz, 2017; Auger et al., 2017; Chrastil, Sherrill, Aselcioglu, Hasselmo, & Stern, 2017; Mao, Kandler, McNaughton, & Bonin, 2017; Vedder, Miller, Harrison, & Smith, 2017; Shine, Valdés-Herrera, Hegarty, & Wolbers, 2016; Auger, Zeidman, & Maguire, 2015; Baumann & Mattingley, 2013; Aggleton, 2010; Iaria, Chen, Guariglia, Ptito, & Petrides, 2007; Wolbers, Weiller, & Büchel, 2004).

Auger et al. (2012; see also Troiani, Stigliani, Smith, & Epstein, 2014) examined features of everyday outdoor landmarks, including their size, visual salience, and whether the landmark had a stable and fixed location. They found that, although the parahippocampal cortex (PHC) processed visual features such as size and salience, the retrosplenial cortex (RSC) responded to landmark permanence, specifically only to landmarks that never moved and were completely fixed in their location. In addition, Auger et al. (2012) showed that self-declared good navigators were more consistent than poor navigators at identifying the most permanent landmarks and had increased engagement of the RSC and the anterior thalamus (AThal), a region heavily connected with the RSC (Jankowski et al., 2013; Vann, Aggleton, & Maguire, 2009), when viewing those items. It has also been possible to decode how many permanent landmarks were in view from functional magnetic resonance imaging (fMRI) activity in the RSC, but not from other brain areas (Auger & Maguire, 2013). This shows that the RSC was not simply engaged by the presence of permanence per se but was mechanistically more nuanced, tracking the specific number of permanent items.

When studying individual outdoor landmarks or environments, fMRI studies, including those described above, have typically used items that are already known to participants from the real world (Tu et al., 2015; Auger & Maguire, 2013; Auger et al., 2012; Baumann, Chan, & Mattingley, 2010; Iaria et al., 2007; Spiers & Maguire, 2006; Wolbers et al., 2004). To understand how an environmental representation develops de novo, Auger et al. (2015) devised a virtual environment that was populated by entirely novel landmarks about which participants had no prior experience. Some of these items moved every time they were seen, whereas others stayed fixed in their locations. When these landmarks were learned during the course of fMRI scanning, Auger et al. (2015) found that the RSC became selectively engaged by nonmoving, permanent landmarks and not those which constantly changed their location. A posterior part of the parieto-occipital sulcus (POS) initially responded to the most memorable landmarks, but as more was learned about them, it switched to instead become engaged by the permanent items. The hippocampus was eventually activated by the permanent landmarks at the end of the learning phase. Also at this point, hippocampal responses to permanent landmarks emerged. Moreover, the hippocampus showed increased functional coupling with the RSC, and activity patterns within the hippocampus mapped onto how much participants knew about where the permanent landmarks were located within the environment. This study shows the significant influence of the RSC and the key contribution of permanent landmarks in the formation of environmental representations.

Overall, these findings suggest that the RSC plays a role in representing landmarks and in particular their inherent permanence. This could be linked to the presence of head direction cells that have been identified within the rodent RSC (Cho & Sharp, 2001; Chen, Lin, Green, Barnes, & McNaughton, 1994), perhaps suggesting a mechanism whereby head direction cell firing is centered upon permanent landmarks and this information is integrated within RSC (Bicanski & Burgess, 2016; Auger et al., 2012). It is notable that the rodent AThal and the subiculum of the hippocampus also contain head direction cells (Taube & Muller, 1998; Taube, 1995; Taube, Muller, & Ranck, 1990) and perhaps form a circuit with the RSC based around permanent environmental features (see also Marchette et al., 2015). Recently, human RSC and AThal have also been shown to contain a head direction signal (Shine et al., 2016).

Jacob et al. (2017) investigated how landmarks influence the brain's computation of head direction in rodents by dissociating local landmarks and global direction in a bidirectionally symmetrical environment. They found that some RSC neurons showed bidirectional firing patterns, which may allow them to mediate both ways between visual landmarks and the global head direction signal. In this way, the RSC could use landmarks to compute head direction and, at the same time, use the head direction signal to compute landmark stability. The authors suggest that this points to a mechanism for associating landmarks to or dissociating them from the head direction signal according to their directional stability and/or their utility for orienting.

This latter study highlights an important point. Given that stable environmental cues are normally the most useful for orienting (Epstein & Vass, 2014; Galati et al., 2010), it is unclear what precisely the RSC is processing—the permanent landmarks themselves or the orienting relevance of these useful landmarks. In all previous fMRI studies, these two features have been conflated—stable landmarks were always useful for orienting. This issue needs to be resolved if we are to truly understand the mechanisms at play within the RSC and how this contributes to the formation of environmental representations that permit navigation.

Auger et al. (2012) had participants rate the navigational utility of real-world outdoor items, as well as their permanence, size, and visual salience. Interestingly, in a factor analysis they found that this navigational utility feature loaded on a separate factor than permanence and one that did not evoke RSC engagement. Moreover, there was no difference between good and poor navigators in their ratings of navigational utility, whereas for ratings of permanence the two groups diverged, as described above. This subjective finding seems to link permanence rather than navigational utility to the RSC, although clearly more objective evidence is required.

Consequently, here we investigated whether RSC codes for the absolute permanence of landmarks or if it is in fact responsive to landmarks that can be used for localizing targets. We dissociated these features using a two-by-two factorial design where landmarks were either permanent or transient and were either relevant or irrelevant for finding a treasure chest. We used novel landmarks (from Auger et al., 2015) about which participants had no prior knowledge. Participants learned about the landmarks and were then tested about their landmark knowledge during fMRI scanning.

**Figure 1.**
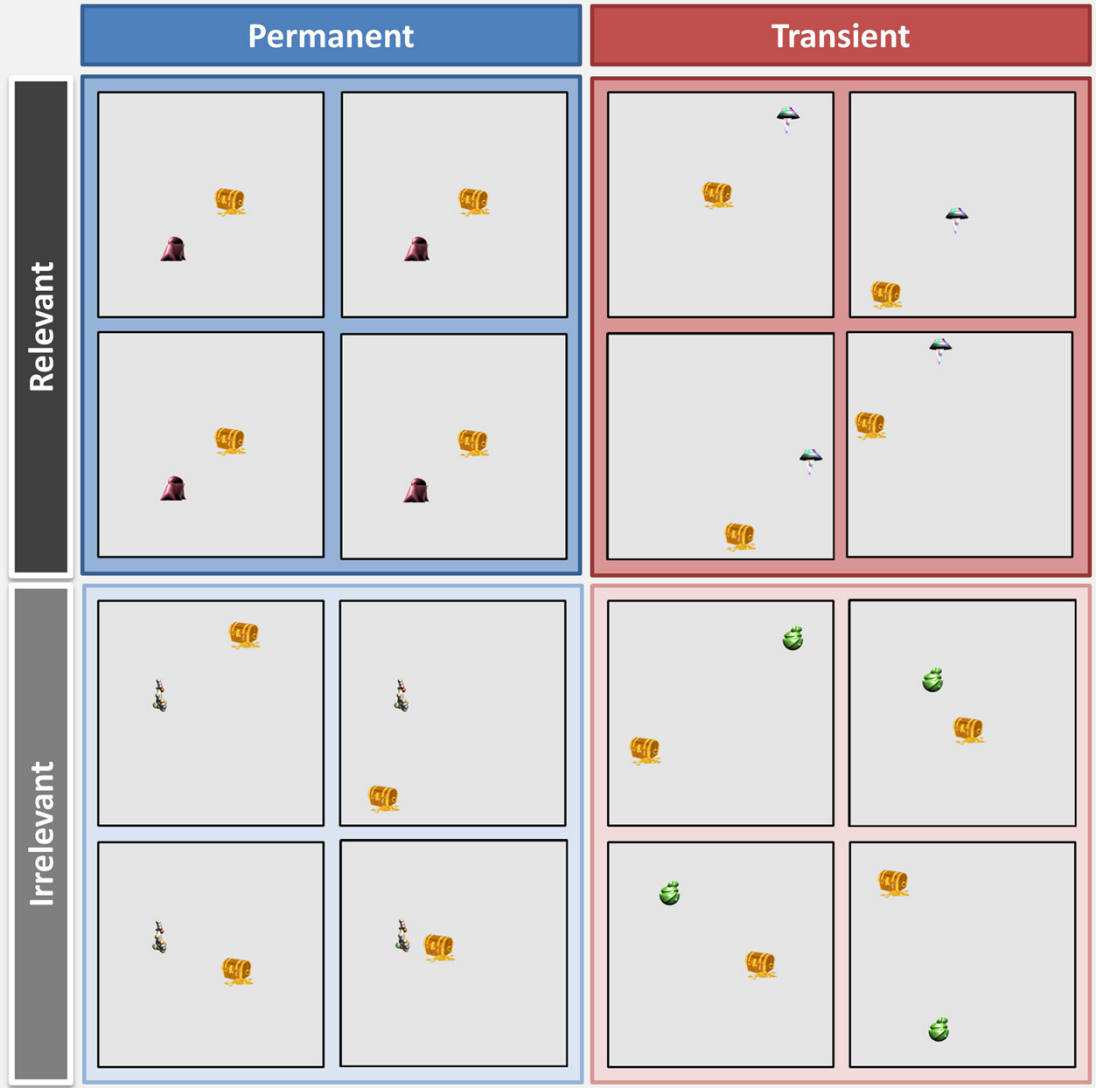
The four experimental conditions. Landmarks varied in terms of their permanence and orienting relevance. For each condition, four example computer screens are shown to represent four different occasions when this stimulus was presented during learning. Permanent landmarks (left/blue) were always positioned in the exact same screen location. Transient landmarks (right/red) appeared in a different place every time. Relevant landmarks (top/darker) could always be used to locate where a treasure chest would be, whereas irrelevant landmarks (bottom/lighter) could not be used to locate the treasure chest.

The nature of our experimental manipulations required participants to have a broad survey-type overview of the positions of landmarks and treasure chests. This would be difficult to achieve in a large-scale environment. Therefore, to enable learning about a sufficient number of landmarks in the prescan phase, the stimuli were presented in locations on a computer screen (Figure 1). This small-scale context was qualitatively different from most previous work examining landmark permanence, where large-scale environments were used. However, given that RSC permanence responses were previously first demonstrated in relation to single isolated objects on a computer screen (Auger et al., 2012), albeit real-world items, we predicted the current approach would yield responses in RSC. That said, it would still be an important proof-of-principle to establish whether representations of landmark permanence or relevance for orienting could be detected in this small-scale setting. This could have an additional benefit, because a simplified desktop version of tasks previously performed in complex environments would be more useful for testing patient populations and perhaps even non-humans. Our prediction, based on the limited subjective evidence described earlier, was that RSC would be responsive to landmark permanence irrespective of orienting value.

This paradigm allowed us to address two additional issues. First, the hippocampus has been shown to process the distance between specific spatial locations (Sherrill et al., 2013; Baumann, Chan, & Mattingley, 2012; Morgan, MacEvoy, Aguirre, & Epstein, 2011; Spiers & Maguire, 2007). RSC has also been implicated in computing distance to a goal location (Sherrill et al., 2013; Baumann & Mattingley, 2010; Wolbers & Büchel, 2005). These previous experiments have generally examined representations of distance coding while people actively navigate within large-scale, complex environments. Using the “relevant” landmarks in our study, namely those which could be used to locate a treasure chest, meant we could investigate whether the RSC and/or hippocampus processed the distance between a relevant landmark and its associated target treasure location on a much smaller scale—the space on a computer screen with people merely viewing landmarks in isolation. The second additional point we could examine related to individual differences. RSC responses to landmark permanence have also been linked to a person's ability to navigate and acquire new spatial information (Auger et al., 2012, 2017; Auger & Maguire, 2013). AThal has also been shown to process permanent landmarks differently, depending on a person's spatial abilities (Auger et al., 2012). We therefore also examined how differences in the amount people learned about the landmarks related to activity within their RSC and its interactions with other connected brain regions, especially those within the head direction circuit, such as AThal and the subiculum of the hippocampus (Shine et al., 2016; Yoder et al., 2011).

## METHODS

### Participants

Thirty-two healthy, right-handed participants took part in the experiment (16 women, mean age = 21.5 years, *SD* = 3.8). All had normal vision and gave written informed consent in accordance with the approval of the local research ethics committee. None had taken part in previous experiments involving these stimuli.

### Stimuli

In a prescan learning session, participants were shown numerous images, one at a time, on a computer screen. Each image contained a single landmark and a treasure chest (see Figure 1 for examples). The landmarks came from the set of unique, novel items, which had been created for previous experiments (Auger et al., 2015, 2017) and of which the participants had no prior experience. The landmarks and treasure chests were viewed multiple times and could appear in any 1 of 64 screen locations (in an 8 × 8 grid arrangement). Each landmark varied according to two key features: (1) Permanence—a landmark either always appeared in the exact same location on the screen on every occasion it was presented (“permanent”) or in a different place every time (“transient”); (2) Relevance—a landmark was either “relevant” for locating the treasure chest (and always appeared in the exact same location relative to a treasure chest) or “irrelevant” for orienting to the treasure chest (the landmark and treasure chest were in completely different relative locations every time they appeared).

This gave rise to four different types of landmarks (Figure 1): Permanent Relevant (both the landmark and treasure chest always appeared in the exact same location on the screen whenever they were seen), Transient Relevant (the landmark and treasure always appeared in different locations on the screen whenever they were seen, but their location relative to each other was fixed), Permanent Irrelevant (the landmark always appeared in the same location whenever it was seen, but the treasure chest was in a different place every time), and Transient Irrelevant (both the landmark and treasure appeared in constantly changing locations). There were 15 stimuli in each condition, giving a total of 60 landmarks.

The landmarks in each condition were matched for a number of other perceptual features based on ratings provided in a separate study with a different set of participants (Auger et al., 2015); these included the following: salience—to what extent does this item grab your attention? *1/Not at all* to *5/Very much*, *F*(3, 56) = 0.350, *p* = .8; associations with other items—does this remind you of anything? *Yes/No*, *F*(3, 56) = 0.502, *p* = .7; strength of association with other items—how strongly does it remind you of this? *1/Only slightly* to *5/Very much*, *F*(3, 56) = 0.439, *p* = .7; how likeable the landmark was—how do you feel about this item? *Like/Dislike*, *F*(3, 56) = 0.886, *p* = .5; animateness—does this item look like it could be alive or not? *Alive/Not alive*, *F*(3, 56) = 0.414, *p* = .7; memorableness—memory of having seen the items after answering all other questions about them *Yes/No*, *F*(3, 56) = 0.039, *p* = 1.0. The landmarks were all the same size, and the locations that appeared on the screen were matched so that an equal number from each of the four conditions appeared in all four quarters of the screen. The locations that treasure chests were positioned relative to landmarks were also matched, so that an equal number of treasure chests appeared above/below and left/right of the four different types of landmark.

The experiment comprised two parts: a learning phase outside the MRI scanner, followed by a testing phase while participants underwent fMRI scanning.

### Prescan Learning Phase

Before starting the learning phase, participants had the task explained to them. They were instructed that they had to view the images of landmarks and treasure chests and concentrate on learning the two key features for each landmark, that is, whether or not it was permanent (always appearing in the exact same place each time it was seen) and whether it could be used to find treasure. They were told that the task inside the MRI scanner would require them to use landmarks to help find treasure. No indication was given about precisely how their knowledge of the landmarks would be tested, just that they needed to focus on learning the two key properties for each landmark.

The learning phase had 15 learning sessions. In each session, all of the 60 landmarks were presented (with a treasure chest) once for 3.5 sec without any intertrial interval in a different randomized order to the other learning sessions. At the end of Sessions 2, 4, 6, 8, 10, 12, 14, and 15, there were "mini-test" periods. On each trial in these mini-tests, an image of a single landmark was shown on a gray background for 2 sec in the center of the screen. Separate screens then immediately asked participants to rate the permanence (is this landmark *Permanent/Transient*) and relevance (could you use this landmark to find the treasure *Yes/No*) of that landmark. As soon as they gave their response, a screen showed participants whether it was correct or incorrect for 1sec before moving to the next trial. Each mini-test had eight trials, except for the final mini-test at the end of Session 15, which had four trials. In this way, each of the 60 landmarks was rated once in the mini-tests. This ensured that exposure to all the landmarks in the learning phase was identical. The mini-tests served two main purposes—they ensured participants remained focused on learning the two key features of each landmark and allowed us to gauge the amount they had learned throughout the learning phase.

The number of landmarks, learning sessions, and mini-tests used were optimized based on a series of pilot experiments to ensure that people could learn and retain sufficient new information about the permanence and relevance of the landmarks without rendering them too fatigued to proceed to the testing phase in the scanner.

### fMRI Testing Phase

**Figure 2.**
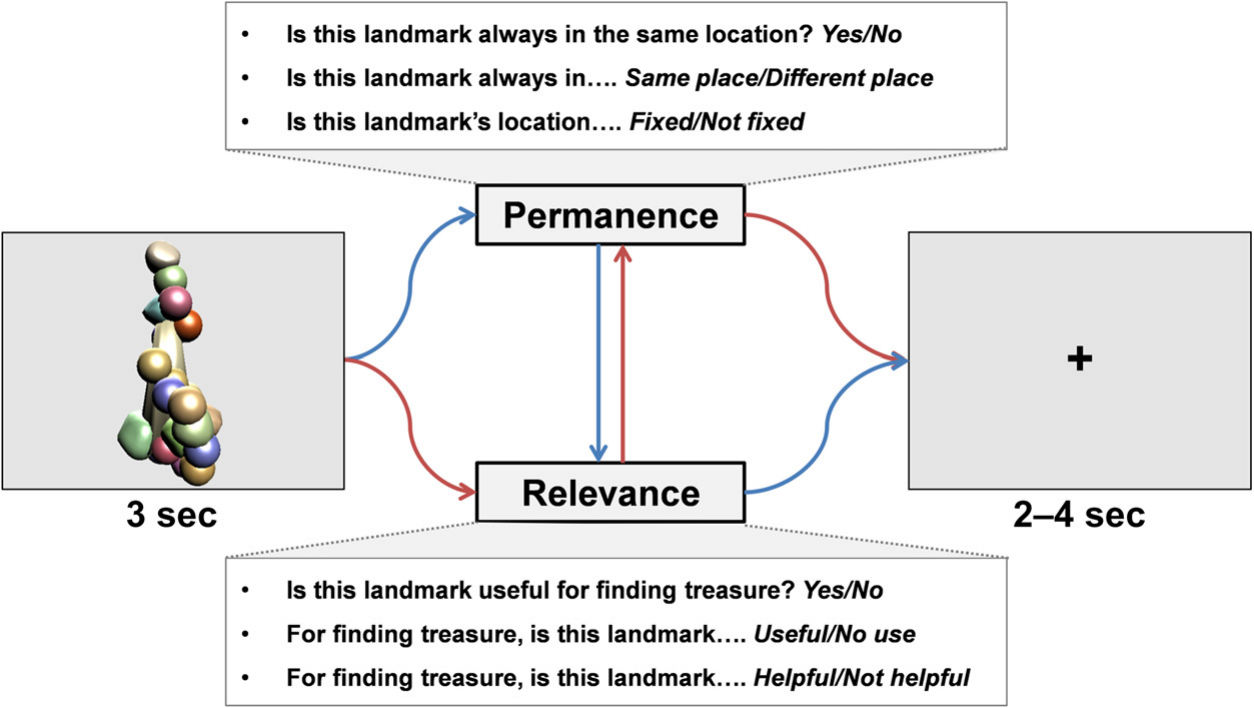
The testing phase during fMRI scanning. An item appeared on the screen, and participants were asked about its permanence and also its relevance for locating the treasure chest. The order of questions was randomized, and the way in which the permanence and relevance questions were asked also varied. There was then a jittered 2- to 4-sec interval before the next trial.

At the end of the learning phase, participants were prepared for fMRI scanning and had the testing phase task explained to them. On each trial, they were presented with an image of a single landmark for 3 sec in the center of the screen on a gray background (Figure 2). Immediately after viewing this landmark image, they rated the permanence and relevance of that landmark before moving on to the next trial. Between trials, there was a 2- to 4-sec jittered interval in which a small black cross was presented in the center of a gray background. Participants were instructed to fixate on this cross during the intertrial interval. The order that the participants were asked to rate the permanence and relevance of landmarks was randomized to ensure they could not anticipate which feature they would need to consider first while the landmark image was on screen. The way in which the permanence and relevance questions were asked also varied to keep participants attending carefully; there were three varieties for each feature: Permanence—(1) Is this landmark always in the same location? *Yes/No*, (2) Is this landmark always in….*Same place/Different place*, (3) Is this landmark's location…*Fixed/Not fixed*; Relevance—(1) Is this landmark relevant for finding treasure? *Yes/No*, (2) For finding treasure, is this landmark…*Useful/No use*, (3) For finding treasure, is this landmark…*Helpful/Not helpful*.

Participants rated the permanence and relevance of each landmark on three separate occasions, using each of the three question variations once, in a randomized order. This gave a total of 180 trials (60 landmarks each rated three times), which were split into three scanning runs of 60 trials each. Within each scanning run, every landmark was viewed and then rated once, in a randomized order.

### Scanning Parameters

T2*-weighted echo-planar images with BOLD contrast were acquired on a 3-T whole-body MRI scanner (Magnetom TIM Trio, Siemens Healthcare, Erlangen, Germany) operated with the standard radio frequency transmit body coil and a 32-channel head receive coil. Scanning parameters were selected to achieve whole-brain coverage but optimized for the hippocampus and surrounding tissue: 48 oblique axial slices angled at −45° from the axial to coronal plane (as defined in Weiskopf, Hutton, Josephs, & Deichmann, 2006), 2.5 mm thickness (with interslice distance factor 20%), repetition time = 3.36 sec (slice repetition time = 70 msec), excitation flip angle = 90°, echo time (TE) = 30 msec, in-plane resolution = 3mm × 3mm, field of view = 192 mm × 192 mm, 64 × 64 matrix, phase encoding in the anterior–posterior direction, 13% oversampling in the phase encoding direction, echo spacing = 500 μsec. For reduction of signal loss in the hippocampal region, slices were angulated, and a *z*-shim gradient moment of +0.6 mT/m msec was applied (Weiskopf et al., 2006). To allow for T1 equilibration effects, the first six “dummy” volumes from each scanning run were discarded. Field maps were acquired using a standard manufacturer's double-echo gradient-echo field map sequence (short TE = 10 msec, long TE = 12.46 msec; 64 axial slices with 2 mm thickness and 1 mm gap yielding whole-brain coverage; in-plane resolution = 3 mm × 3 mm). A 3-D MDEFT T1-weighted structural scan (Deichmann, Schwarzbauer, & Turner, 2004) was acquired for each participant with 1 mm isotropic resolution.

### MRI Data Preprocessing

fMRI data were analyzed with SPM8 (www.fil.ion.ucl.ac.uk/spm). Images were bias-corrected, realigned, unwarped (using the field maps), and normalized to a standard EPI template in MNI space with a resampled voxel size of 3 × 3 × 3 mm. For all the whole-brain univariate and connectivity analyses, images were then smoothed using a Gaussian kernel with FWHM of 8 mm. For multivoxel pattern analysis (MVPA), unsmoothed images were used to facilitate the detection of information present across patterns of voxels.

### Behavioral Analyses

We compared the rates that participants learned landmark permanence and orienting value during the prescan learning phase. This was achieved by performing separate linear regression analyses for participants' learning of landmark permanence and orienting relevance and then directly comparing the slopes with a *t* test. We also compared the accuracy of participants' ratings of permanence and orienting relevance in the test phase within the scanner. In the testing phase task, landmark permanence and orienting relevance was asked in three different ways (Figure 2). The ordering of the permanence/relevance questions was also randomized. We therefore additionally analyzed the responses using one-way ANOVAs to determine whether the way or order in which the permanence and relevance questions were asked had any impact on the accuracy of responses. A threshold of *p* < .05 was used throughout. All statistical analyses were performed using SPSS version 20 (SPSS, Inc., Chicago, IL).

### Whole-brain fMRI Univariate Analyses

To assess fMRI responses in relation to the permanence and orienting value of landmarks across the whole brain, we first performed an interaction analysis. The main effects of each condition (landmark permanence and orienting relevance) were then analyzed. Namely, we compared whole-brain BOLD responses for permanent versus transient, transient versus permanent, relevant versus irrelevant, and irrelevant versus relevant landmarks. For each contrast, we created regressors for each condition of interest and convolved them with the canonical hemodynamic response function.

Each testing phase trial was modeled from the time of onset of a landmark image for 1.5 sec (the first half of the landmark image presentation time) given previous findings suggesting that this time frame ensures responses corresponded to automatic and rapid processing of landmarks (Auger et al., 2012). Separate participant-specific movement regressors were treated as covariates of no interest. Subject-specific parameter estimates pertaining to each regressor of interest (betas) were calculated for each voxel. Second-level random effects analyses were then run using one-sample *t* tests on the parameter estimates (collapsing across the three scanning runs of the task). We also performed additional univariate analyses, which included regressors at the second level relating to each individual's accuracy in answering questions about landmark features. For all contrasts, we report any activation that survived a whole-brain family-wise error (FWE)-corrected threshold of *p* < .05, unless otherwise stated.

The hippocampus has been reported to process the distance between specific spatial locations (Sherrill et al., 2013; Baumann et al., 2012; Morgan et al., 2011; Spiers & Maguire, 2007). Therefore, we also looked for BOLD responses related to the distance between a landmark and its associated treasure location. For every relevant landmark, we calculated the distance between it and its target treasure location. These values were used to create parametric regressors for a whole-brain general linear model fMRI analysis. Specifically, we looked for activity that was linearly modulated by a target location, which was closer or farther away from its associated landmark. We report any fMRI activations that survived a whole-brain FWE-corrected threshold of *p* < .05, except for the hippocampus where, given our prior hypotheses regarding this specific region, we report activations at a whole-brain uncorrected threshold of *p* < .001.

### Connectivity Analyses

We were also interested in RSC interactions with other brain regions and how this may relate to how well participants had learned information about the landmarks. As such, for any landmark features to which RSC was responsive in the whole-brain univariate analyses (a so-called “feature-of-interest”), we also investigated its interactions with other brain areas and how these interactions varied depending on how well participants had learned that feature. Specifically, we used a generalized psychophysiological interaction (gPPI) analysis (McLaren, Ries, Xu, & Johnson, 2012; Friston et al., 1997) to examine the functional coupling between RSC and the rest of the brain while people viewed landmarks possessing the particular feature-of-interest. We then also added the participants' accuracy scores for that landmark feature during the in-scanner testing phase as a second-level covariate of interest. We performed additional gPPI analyses for any other regions shown to be responsive to landmark permanence or relevance from the whole-brain univariate analysis.

The PPI analyses were performed using the Generalized Form of Context-Dependent Psychophysiological Interactions SPM toolbox (McLaren et al., 2012). As seed regions, we used clusters from the corresponding whole-brain univariate fMRI contrasts with a specific focus on RSC (i.e., the clusters within RSC, which were responsive to a particular feature-of-interest). For all the gPPI analyses, we report any significant activation that survived a whole-brain FWE-corrected threshold of *p* < .05, unless otherwise stated.

For any functional connectivity identified by the gPPI analyses, we used dynamic causal modeling (DCM; Stephan, Penny, Daunizeau, Moran, & Friston, 2009; Friston, Harrison, & Penny, 2003) to investigate the nature of the information flow between the regions. The gPPI analyses specifically indicated which regions increased their interaction in connection with more knowledge of the feature-of-interest. We therefore compared how the nature of the interaction between the regions may differ between participants who had learned the information particularly well and those who had learned less well. To do this, we created a regressor for the feature-of-interest for use in a parametric empirical Bayes (PEB) DCM analysis (Friston et al., 2016).

For each participant, we created a design matrix with two main regressors of interest: one modeling all landmarks, to be used as the input for each DCM model (C matrix), and another for just those with the specific feature-of-interest (e.g., amount of learning about permanent landmarks), to be used as the models' modulatory input (B matrix). Each model assumed the presence of endogenous self-connections and reciprocal connectivity between the two brain regions (A matrix) that emerged from the gPPI analyses. We used DCM12 to fit each model to the fMRI data and also modeled stochastic fluctuations in the state equations to account for neural noise, which is particularly relevant for these endogenously driven interactions (Daunizeau, Stephan, & Friston, 2012).

We created a full PEB model, with all connections mentioned above present (i.e., complete bilateral reciprocal connectivity between the two brain regions), and a second-level regressor for the feature-of-interest identified in the gPPI analysis to act as the modulatory B matrix. We then compared this full PEB model with two nested PEB models. These two nested models each removed one of the two B matrix modulatory inputs so that we could compare the modulatory effect of Region 1 upon Region 2 and vice versa, in relation to the feature-of-interest (Figure 5B shows the precise model architectures that were compared).

It should be noted that gPPI and DCM analyses differ in the precise data that are modeled. DCM models are fit on the main task model, whereas gPPI adds a regressor to the univariate model and is therefore fit on the residuals of the task activity model.

### Multivoxel Pattern Analysis

Previous studies have used MVPA as a potentially more sensitive measure for detecting neural representations compared with mass univariate analyses (e.g., Bonnici et al., 2012). We therefore also used MVPA to examine subject-specific neural representations of the two landmark features (Chadwick, Bonnici, & Maguire, 2012; Haynes & Rees, 2006; Norman, Polyn, Detre, & Haxby, 2006). Separate regressors were created for each of the 180 trials. Participant-specific parameter estimates pertaining to each trial regressor were then calculated and used in the MVPA analyses. We selected ROIs to use for the MVPA analyses from brain areas shown in this and previous studies (Auger & Maguire, 2013; Auger et al., 2012) to process permanence and related landmark features, namely, RSC, hippocampus, and PHC, as well as additional brain regions identified in this study's whole-brain univariate or PPI analyses. ROIs were defined anatomically for RSC, hippocampus, and PHC using bilateral masks, which were delineated by an experienced researcher, not involved in this project, on an averaged structural MRI brain scan from an independent group of participants (*n* = 30) and guided by Duvernoy (1999), Insausti et al. (1998), and Vann et al. (2009). The other areas were defined functionally from the output of the univariate and gPPI analyses.

MVPA analyses were performed for every subject to ascertain whether or not it was possible to decode the type of landmark being viewed based on patterns of activation in each of the ROIs. All MVPA analyses used a linear support vector machine (SVM) implemented through LIBSVM (Chang & Lin, 2011) with fixed regularization hyperparameter *C* = 1. We used a standard cross-validation procedure throughout (Hsu & Lin, 2002; Duda, Hart, & Stork, 2001) whereby a single trial is assigned as the test trial and all others as training trials. In each instance, an SVM was trained using the training trial data. The SVM was then presented with the fMRI data from the test trial to “guess” what type of trial it was (i.e., what type of landmark was being viewed while that specific fMRI activation pattern was elicited). This process was repeated, changing the test trial each time until all trials had been tested once. Classifier accuracy is defined as the proportion of the SVM guesses that were correct.

In line with previous studies (Auger & Maguire, 2013; Bonnici et al., 2012; Chadwick et al., 2012), we used feature selection to first identify the voxels within each ROI, which were most likely to carry relevant information pertaining to what was being classified (Guyon & Elisseeff, 2003). Only the most informative participant-specific voxels within each ROI were then used for a separate final classification to establish a region's classifier accuracy value. This process in effect increases the signal-to-noise ratio. The feature selection used a multivariate searchlight strategy (Kriegeskorte, Goebel, & Bandettini, 2006). This aims to determine the information present within a “local environment” surrounding each voxel within an ROI. We used a series of classifications (as described above) to assess the amount of relevant information present within a sphere (of radius 3 voxels) surrounding each voxel within the ROIs. Only voxels with responses carrying the most information were then selected for use in the final classification.

To avoid any form of circular analysis, we used independent data sets for the feature selection and final classifications (Kriegeskorte, Simmons, Bellgowan, & Baker, 2009). The testing phase consisted of three scanning runs; we therefore used two runs for feature selection and the independent data set from the remaining run for the final classification. This was repeated twice more, changing the scanning run that was used for the final classification on each occasion. The classifier accuracy values from these three repetitions were then averaged to provide an overall threefold cross-validation. This produced a single participant-specific classification accuracy value for each ROI. We then performed *t* tests on these values to assess whether or not accuracy across all subjects was significantly above chance (i.e., *t* tests were all one tailed).

We first used this MVPA procedure to assess the ROIs' response patterns associated with representations of the features with a four-way classification of landmark type (Permanent Relevant vs. Transient Relevant vs. Permanent Irrelevant vs. Transient Irrelevant; chance = 25%). Similar to the gPPI connectivity analysis, we then looked for any relationship between these results and people's knowledge about the landmarks. For ROIs implicated in coding for landmark features by the four-way classification, we also performed separate two-way classifications of permanence and relevance to assess representations of the two properties independently.

Finally, we investigated whether it was possible to classify the distance between a relevant landmark and its associated treasure location. We took a median split of the relevant landmarks' distances from their related treasure location to define “close” and “far” groups. We then performed an MVPA analysis to determine whether the activation patterns elicited while viewing these landmarks might contain information about the proximity of their target location.

## RESULTS

### Behavioral Data

The participants successfully learned both the permanence and relevance of the landmarks, and there was no difference in their accuracy scores for the two features in the final prescan learning phase mini-test (mean permanence accuracy = 93.0%, *SD* = 15.9; mean relevance accuracy = 86.7%, *SD* = 16.8; *t*_31_ = 1.761, *p* = .09). There was also no difference in the rate at which they learned landmark permanence or relevance (mean difference between slopes of the linear learning regressors = 0.05, *SD* = 0.26; *t*_31_ = 1.084, *p* = .3).

Responses made by participants during the in-scanner testing phase task also indicated that there were no differences in how well subjects knew the permanence and orienting relevance of landmarks (mean permanence accuracy = 90.1%, *SD* = 10.7; mean relevance accuracy = 87.7%, *SD* = 12.5; *t*_31_ = 0.426, *p* = .4).

The three different ways in which the questions were asked for the task inside the scanner also had no impact on the accuracy of responses for permanence (question one mean accuracy = 90.6%, *SD* = 9.8; question two mean accuracy = 89.2%, *SD* = 12.2; question three mean accuracy = 90.4%, *SD* = 10.6; *F*(2, 93) = 0.155, *p* = .9) or relevance (question one mean accuracy = 87.6%, *SD* = 13.2; question two mean accuracy = 87.3%, *SD* = 13.0; question three mean accuracy = 88.3%, *SD* = 11.9; *F*(2, 93) = 0.054, *p* = .9).

The order of the questions also had no impact on the accuracy of participant responses for either permanence (mean accuracy if first question = 89.7%, *SD* = 11.1; mean accuracy if second question = 90.5%, *SD* = 10.3; *t*_31_ = 0.324, *p* = .8) or relevance (mean accuracy if first question = 88.0%, *SD* = 13.4; mean accuracy if second question = 87.2%, *SD* = 12.0; *t*_31_ = 0.132, *p* = .9). Overall, therefore, any differences in fMRI responses could not be attributed to disparity in the extent that participants knew the two landmark features or how this information was elicited.

### Areas Responding to Properties of the Landmarks across the Whole Brain

We first performed whole-brain univariate contrasts to look for regions that were more engaged by permanent and/or relevant landmarks. An interaction analysis found no regions where activity was influenced by a combination of both landmark properties. We then performed separate analyses to assess the main effects of each individual condition.

**Figure 3.**
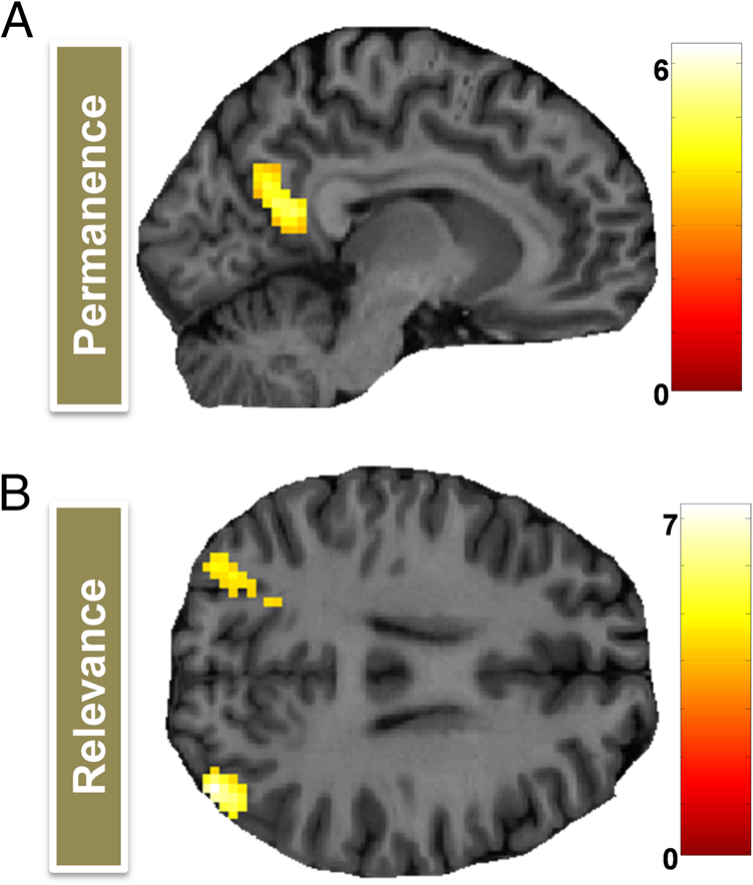
Brain areas responsive to landmark permanence and relevance—whole-brain univariate analysis. (A) The RSC and posterior parts of POS were more engaged by permanent than transient landmarks. (B) Bilateral clusters in the angular gyrus were more active when people viewed a relevant than an irrelevant landmark. Activations are displayed on a sagittal (A) and axial (B) section of a single representative participant's structural MRI brain scan. The color bars indicate each voxel's associated *Z* score.

Comparing fMRI responses when participants viewed permanent and transient landmarks, there was increased activity for permanent items within the right RSC (Figure 3A; 15, −52, 19, *Z* = 5.86; the left RSC was also active just below threshold), which extended into posterior parts of the POS, as well as additional bilateral clusters in posterior occipital cortex (left: −18, −88, −8, *Z* = 6.34; right: 18, −91, −2, *Z* = 6.31). A contrast comparing landmarks relevant for localizing the treasure chest with those which were irrelevant produced no significant activation in RSC, but there were bilateral clusters of activation in the region of the angular gyrus (Figure 3B; right: 39, −82, 25, *Z* = 7.26; left: −33, −82, 34, *Z* = 6.05). No brain areas were more responsive to transient than permanent landmarks or irrelevant than relevant landmarks. No brain regions were responsive to the accuracy of answering the questions about landmark features.

**Figure 4.**
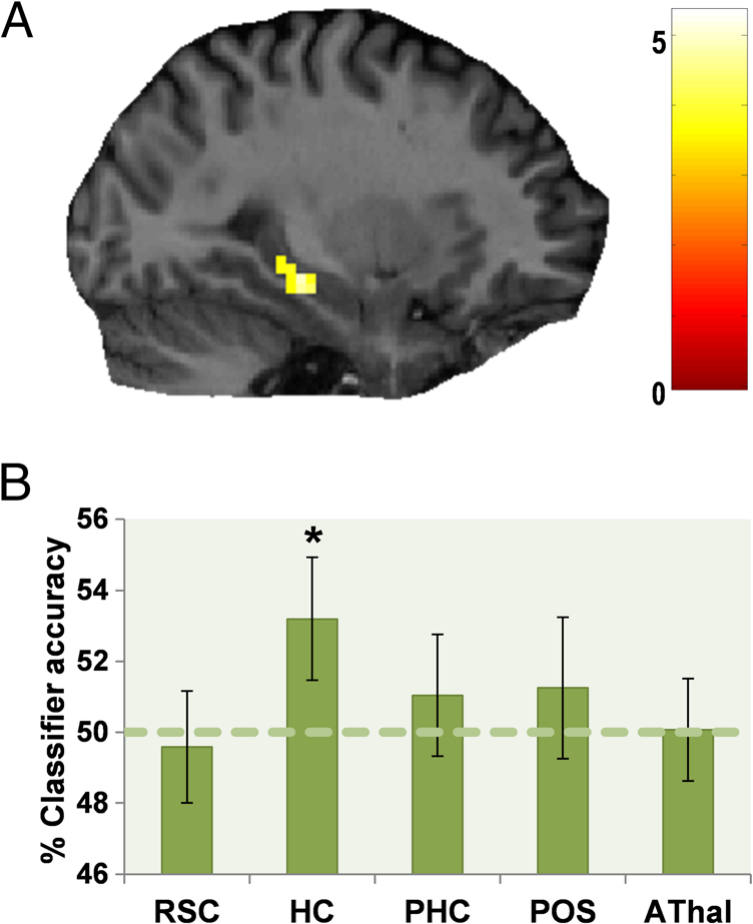
Hippocampal processing of distance to the treasure and orienting relevance. (A) The hippocampus increased its engagement for landmarks that were associated with a more distant target treasure location. Activations are displayed on sagittal sections of a single representative participant's structural MRI brain scan. The color bar indicates each voxel's *Z* score. (B) Only the hippocampus had patterns of fMRI activity, which could be used to decode whether a relevant landmark's associated treasure was nearby or farther away. The green dashed line indicates the chance level (50%) for this two-way classification; error bars show the *SEM*, and * denotes classifications that are significantly above chance (*p* < .05). RSC = retrosplenial cortex; HC = hippocampus; PHC = parahippocampal cortex; POS = parieto-occipital sulcus; AThal = anterior thalamus.

For relevant landmarks, we also looked for fMRI responses related to the distance between them and their associated treasure location. A larger distance between a landmark and its target location was associated with a greater BOLD response in right hippocampus (Figure 4A; 30, −28, −11, *Z* = 4.89). No region was more engaged by landmarks associated with closer treasure locations.

### RSC Interactions with Other Brain Areas and the Effect of Landmark Knowledge

**Figure 5.**
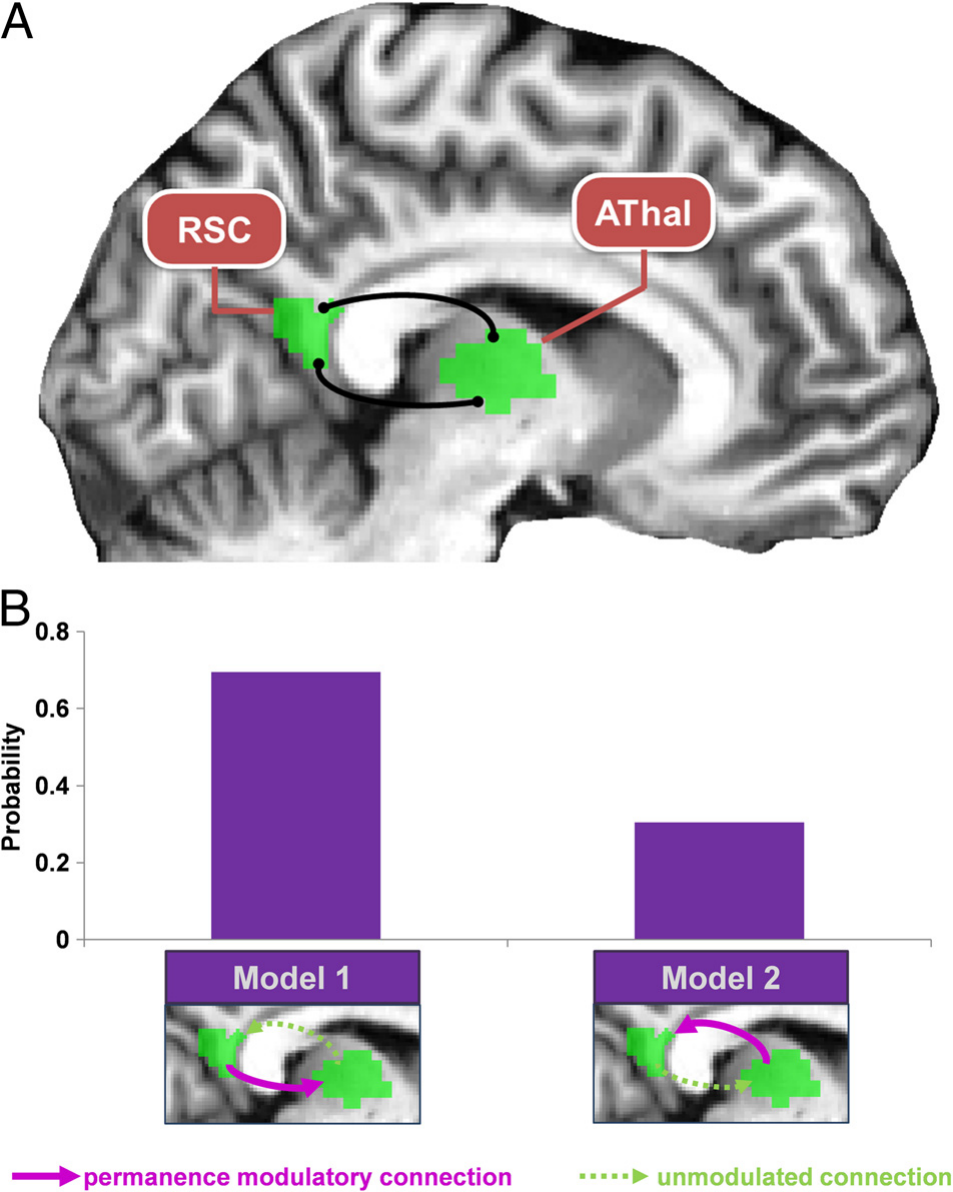
RSC connectivity associated with individual differences in permanence knowledge. RSC = retrosplenial cortex; AThal = anterior thalamus. (A) A gPPI analysis showed that, when participants viewed an image of a permanent landmark, the better they had learned landmark permanence, then the more their RSC interacted with AThal. (B) To examine the nature of this RSC–AThal interaction in relation to learning of landmark permanence, we performed a PEB DCM analysis. The winning model (Model 1) indicates that RSC drove activity in AThal in accordance with individuals' learning of landmark permanence.

We then looked for brain areas with which the permanence-responsive RSC was interacting and how this may be related to how well participants had learned landmark permanence. An initial gPPI analysis across the whole participant group found no significant interaction between regions. However, a further gPPI analysis, which took into account individuals' learning of landmark features, revealed that, when participants viewed permanent landmarks, the better they had learned the landmark permanence, then the more their RSC displayed functional coupling with the AThal (−12, −19, 7, *Z* = 5.80; Figure 5A). There was also an additional significant cluster in the cerebellum (the posterior part of the quadrangular lobe: −9, −70, −11, *Z* = 6.59). At a reduced threshold (*p* < .001 whole brain uncorrected), there was also increased activity in the left hippocampus, including the subiculum (−21, −22, −8, *Z* = 4.34). This is particularly interesting, given that the RSC, AThal, and subiculum are known to contain head direction cells in rodents (Sharp, Blair, & Cho, 2001). Further gPPI analyses were run using the other brain areas shown to be responsive to landmark permanence or relevance in the whole-brain univariate analyses (as described above). No other region displayed any differences in functional connectivity relating to the amount of information learned about landmarks.

Next, we investigated the nature of the RSC–AThal interaction using DCM. We compared a full PEB model of connectivity between the two regions with two nested PEB models. The first model excluded the connection for individuals' permanent landmark learning from AThal to RSC, and the second excluded the modulatory connection in the opposite direction. Model 1 therefore represented the effect of RSC driving responses in AThal according to how well participants had learned about permanent landmarks, and Model 2 represented the effect of AThal driving responses in RSC in line with the level of permanence learning (see Figure 5B). Model 1 was the clear winner (Model 1 posterior probability = .695, Model 2 posterior probability = .305). This indicates that the increased interaction between RSC and AThal revealed by the gPPI analysis likely reflected an increase in RSC driving responses in AThal, the better people knew the permanence of landmarks.

### Multivoxel Pattern Analysis

**Figure 6.**
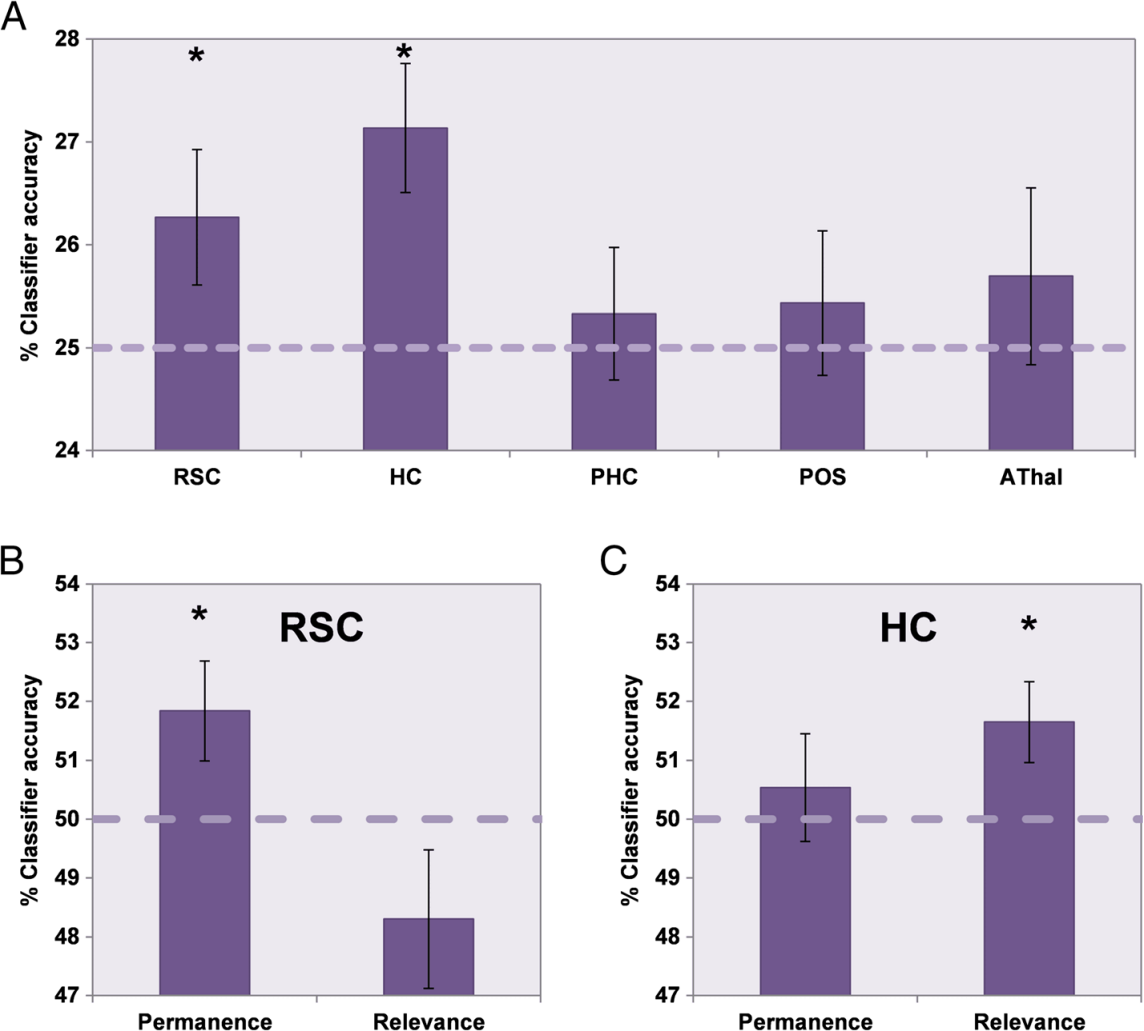
MVPA analysis of landmark permanence and relevance. (A) The classification accuracy for decoding between the four types of landmark in each of the ROIs. RSC = retrosplenial cortex; HC = hippocampus; PHC = parahippocampal cortex; POS = parieto-occipital sulcus; AThal = anterior thalamus. Above chance classification was only possible for RSC and hippocampus. To determine which feature each region was particularly sensitive to, additional two-way classifications of landmark permanence and relevance were performed in RSC (B) and hippocampus (C). RSC responses could be used to classify landmark permanence but not relevance, whereas hippocampal activity could be used to classify orienting relevance but not permanence of landmarks. Dashed lines indicate each classification's chance level, error bars show the *SEM*, and * denotes classifications that are significantly above chance (*p* < .05).

To explore the representations of landmark features in greater detail, we used MVPA. We first investigated whether it was possible to decode which of the four landmark types (Permanent Relevant, Transient Relevant, Permanent Irrelevant, or Transient Irrelevant) a participant was viewing based on the multivoxel patterns of activity in RSC, hippocampus, and PHC (defined anatomically), as well as the two other regions implicated in this study—the POS (the parts of the functional cluster responding more to permanent than transient landmarks in the univariate analysis, see Figure 3A, excluding the parts in RSC) and AThal (as found in the PPI analysis; see Figure 5A). Figure 6A illustrates results of this four-way MVPA analysis. Landmark type could be classified above chance (25%) based on responses in RSC (mean accuracy = 26.3%, *SD* = 3.7; *t*_31_ = 1.924, *p* = .03) and hippocampus (mean accuracy = 27.1%, *SD* = 3.5; *t*_31_ = 3.410, *p* < .001), but not in PHC (mean accuracy = 25.3%, *SD* = 3.6; *t*_31_ = 0.512, *p* = .3), POS (mean accuracy = 25.4%, *SD* = 4.0; *t*_31_ = 0.618, *p* = .3), or AThal (mean accuracy = 25.7%, *SD* = 4.9; *t*_31_ = 0.808, *p* = .2).

To establish whether the significant four-way classification in RSC and hippocampus was driven by representations of landmark permanence or relevance, we performed independent two-way classifications for each feature based on responses in the two regions. A 2 × 2 ANOVA comparing classifier accuracy in RSC and hippocampus for permanence and relevance demonstrated a significant interaction, *F*(1, 31) = 7.550, *p* = .01. *t* tests demonstrated that, in RSC (Figure 6B), response patterns could be used to classify the permanence (mean accuracy = 51.8%, *SD* = 4.8; *t*_31_ = 2.168, *p* = .02) but not relevance (mean accuracy = 48.3%, *SD* = 6.7; *t*_31_ = −1.444, *p* = below chance classification) of landmarks significantly above chance. The opposite was true of hippocampus (Figure 6C), where relevance (mean accuracy = 51.6%, *SD* = 3.9; *t*_31_ = 2.398, *p* = .01) but not permanence (mean accuracy = 50.5%, *SD* = 5.2; *t*_31_ = 0.586, *p* = .3) could be decoded. We also performed post hoc two-way classifications in PHC, POS, and AThal for both permanence and orienting relevance. Unsurprisingly, given the results of the four-way classification reported above, it was not possible to classify either feature in any of the regions with above chance accuracy.

Similar to the univariate analysis of fMRI responses relating to the distance between a relevant landmark and its associated treasure location, we also examined whether it was possible to decode this distance using MVPA. The multivoxel response pattern elicited in hippocampus while people viewed an image of a landmark could be used to classify whether or not it was relevant for finding treasure that was nearby or farther away (Figure 4B; mean accuracy = 53.2%, *SD* = 9.8; *t*_31_ = 1.852, *p* = .04). This was not the case in the other ROIs (RSC mean accuracy = 49.6%, *SD* = 8.9; *t*_31_ = −0.264, below chance classification; PHC mean accuracy = 51.0%, *SD* = 9.7; *t*_31_ = 0.605, *p* = .3; POS mean accuracy = 51.3%, *SD* = 11.3; *t*_31_ = 0.626, *p* = .3; AThal mean accuracy = 50.1%, *SD* = 8.2; *t*_31_ = 0.048, *p* = .5).

## DISCUSSION

The RSC has been linked with the processing of permanent landmarks in a number of previous studies (Auger et al., 2012, 2015, 2017; Marchette, Vass, Ryan, & Epstein, 2014; Troiani et al., 2014; Auger & Maguire, 2013). However, it is unclear whether these RSC responses truly reflect representations of a landmark's permanence or instead the fact that permanent landmarks tend to be the most useful cues for orienting. Here we dissociated these two important landmark properties. When people viewed permanent landmarks, there was increased activity in RSC extending posteriorly into the POS. The same was not true, however, for landmarks that could be used to locate target treasure chests. We also found that RSC contained subject-specific patterns of activity associated with knowledge of permanence, but not relevance, of landmarks. This was not the case for any other brain region. In contrast, the orienting relevance of landmarks was associated with engagement of the angular gyrus and the hippocampus, with the hippocampus also representing the distance between a treasure chest and its associated landmark. These distinct patterns of brain activity associated with landmark permanence and orienting relevance could not be attributed to differences in the rate at which the two features were learned or the overall amount of information that was acquired. Therefore, a landmark's permanence seems to be a primary feature processed by RSC, with orienting value of landmarks and their spatial relationships coded by other regions.

### Landmark Permanence

RSC has been implicated in a diverse range of complex cognitive functions including navigation, scene processing, episodic memory, and imagination of future and fictitious events (Vann et al., 2009; Spreng, Mar, & Kim, 2009; Epstein, 2008). However, there is limited evidence to indicate what specific role it might contribute to these processes. We have previously proposed that a key function of the RSC is to identify permanent stable landmarks, which might then be used to build environmental representations (Auger et al., 2012, 2015, 2017; Auger & Maguire, 2013). However, the permanent landmarks in these previous experiments were always inherently more relevant and reliable cues for orienting. Here, by dissociating these two properties, we were able to confirm that RSC does indeed appear to primarily process landmark permanence independent of any utility for making spatial judgements. This accords with our previous factor analysis finding that navigational utility loaded onto a separate factor than permanence and did not evoke RSC engagement (Auger et al., 2012). Moreover, in the same study, there was no difference between good and poor navigators in their ratings of navigational utility, whereas poor navigators were significantly less consistent in rating permanence.

Previous work has demonstrated that experiencing landmarks from first-person and survey-type perspectives can give rise to differences in the associated neural responses (Boccia, Guariglia, Sabatini, & Nemmi, 2016; Boccia, Nemmi, & Guariglia, 2014; Shelton & Gabrieli, 2002). The current survey-type paradigm is qualitatively different from previous first-person perspective work, which related RSC activity to landmark permanence (Auger et al., 2012, 2015). Nevertheless, we found that RSC was specifically responsive to the permanence of landmarks even on this small scale. This suggests that the scope of RSC permanence processing may be broad, encompassing situations involving items in space in different frames of reference. Our results also indicate that tasks need not involve complex virtual spaces to study the RSC, which could be useful for testing patients and perhaps nonhumans.

The dominance of permanence, rather than relevance, representations in RSC and POS seems to be inconsistent with the suggestion that a key function of these regions lies in using landmarks to localize and orientate within space (Epstein & Vass, 2014). The role RSC plays in these more complex processes could merely reflect the fact that they are usually centered upon permanent environmental features. RSC has also been suggested to assist in translating between and integrating egocentric and allocentric spatial information (Sherrill et al., 2013; Sulpizio, Committeri, Lambrey, Berthoz, & Galati, 2013; Vann et al., 2009; Byrne, Becker, & Burgess, 2007), but again this could similarly reflect the reliance of these processes on manipulating mainly permanent cues.

RSC has also been implicated in processing information relating to the distance and direction to a goal location (Vedder et al., 2017; Sherrill et al., 2013; Baumann & Mattingley, 2010; Wolbers & Büchel, 2005). However, this did not appear to be the case in this study; only the hippocampus showed any sensitivity to the distance between a landmark and its associated treasure location. This may relate to our use of small-scale space and not navigation within an environment or the recent finding that goal proximity coding switches from hippocampus to RSC over time (Patai et al., preprint). It could also be the case that the RSC's apparent involvement in these processes in previous studies may in fact have reflected their reliance upon the use of permanent, stable environmental cues. From the perspective of navigation system robustness, it may be advantageous that the RSC responds specifically to permanence, as that is perhaps less likely to change than orienting relevance.

### Orienting Relevance

The hippocampus has been found to be responsive to permanent landmarks (Auger et al., 2015). This might seem at odds with the current result where there was no evidence of hippocampal activity relating to landmark permanence. However, the reason for this may be because the hippocampus in that previous study was observed to process permanent landmarks when they were associated with a precise location (Auger et al., 2015) and not for landmarks that were devoid of specific spatial connections (Auger & Maguire, 2013; Auger et al., 2012). This points to the hippocampus perhaps playing a role in processing the spatial relationships between landmarks and other parts of the environment, rather than the permanence of landmarks themselves. This is consistent with the results of this study, where a landmark's permanence was dissociable from other spatial relationships and consequently the hippocampus was not engaged.

Further weight is added to this account of hippocampal function by the fact that the hippocampus was responsive to the distance between a landmark and its related treasure. This accords with previous reports of the hippocampus representing the distances between items in larger, three-dimensional environments (Sherrill et al., 2013; Baumann et al., 2012; Morgan et al., 2011; Spiers & Maguire, 2007). Here we demonstrate that this also holds true for smaller-scale spatial relationships. But there may be other explanations for this finding. Hippocampal engagement here could merely reflect associative binding between a landmark and a treasure chest, although we believe this is unlikely given that the same binding was involved for treasure that was near and farther away and yet it was possible to differentiate the two from activity within the hippocampus. Another consideration is the nature of the treasure chest. Although each trial contained a unique landmark, the treasure chest was the same across trials, although it had a different connotation in each case, and participants knew this and clearly performed well at learning orienting relevance. Nevertheless, it could be argued that the distance effects we noted were related to the amount of pattern separation (Leutgeb, Leutgeb, Moser, & Moser, 2007) that was required, with near and farther away treasure chests being perhaps more easily distinguishable. However, this cannot explain the main MVPA finding where it was possible to decode whether landmarks were relevant or not for orienting. It is worth noting that, although the MVPA effect sizes were small, it was nevertheless possible to achieve significant above-chance classification. The magnitude of classifier accuracy depends on various elements of an experimental paradigm (as described by Chadwick et al., 2012). Here the requirement for whole-brain coverage precluded the use of high-resolution scanning, which may have yielded a greater degree of classifiable information.

We also found the angular gyrus was responsive to the orienting relevance of landmarks. This brain region has been posited to play a role in a broad array of cognitive functions (Seghier, 2013), from reading, comprehension, and number processing (Arsalidou & Taylor, 2011; Houdé, Rossi, Lubin, & Joliot, 2010; Price & Mechelli, 2005) to social cognition (Mar, 2011). As such, the angular gyrus response in this study to landmarks with relevance for orienting could be interpreted in several ways. Knowledge of a landmark's relevance for orienting required linking this semantic concept to its visual attributes, and this type of semantic processing has been consistently attributed to angular gyrus (Binder, Desai, Graves, & Conant, 2009; Vandenberghe, Price, Wise, Josephs, & Frackowiak, 1996), particularly for concrete rather than abstract concepts (Wang, Conder, Blitzer, & Shinkareva, 2010). Angular gyrus is also implicated in shifting attention to stimuli with a particularly salient value or meaning (Studer, Cen, & Walsh, 2014; Taylor, Muggleton, Kalla, Walsh, & Eimer, 2011; Gottlieb, 2007), which the relevant landmarks could certainly be said to have possessed. A third interpretation is that angular gyrus was activated because of its role in conflict resolution between inputs (Nee, Wager, & Jonides, 2007; Fan, Flombaum, McCandliss, Thomas, & Posner, 2003), perhaps the task of resolving the conflicting permanence and relevance information engaged angular gyrus. This final interpretation is more problematic given that the level of “conflict” between the landmarks' properties would not necessarily be greater among the relevant than nonrelevant landmarks; each landmark had the same two binary conflicting attributes. That said, it is difficult to conclusively distinguish whether the angular gyrus response to landmarks with relevance for orientation reflected generalized semantic processing, more specific direction of visuospatial attention, or the resolution of conflicting landmark properties. In truth, it may be some combination of the three.

### Interindividual Differences in Landmark Knowledge

Responses in RSC also related to how well people had learned about landmark permanence. Better learning about permanence was associated with more differentiable response patterns in RSC. Furthermore, the better participants had learned which landmarks were permanent, the more their RSC interacted with AThal while viewing those landmarks.

Previous studies have demonstrated that activity in RSC is related to a person's ability to acquire new spatial information and to navigate (Auger et al., 2012, 2017; Auger & Maguire, 2013). In each case, variation in these general spatial abilities was also found to be associated with specific differences in processing landmark permanence, both behaviorally and in fMRI responses in RSC. This study adds to this growing body of evidence that performance in some spatial tasks may be directly linked to RSC permanence representations both of highly familiar everyday items (Auger & Maguire, 2013; Auger et al., 2012) as well as while learning new information about previously unfamiliar landmarks (Auger et al., 2015, 2017).

RSC does not act in isolation and is connected to a wide range of other cortical and subcortical brain regions (Sugar, Witter, van Strien, & Cappaert, 2011; Greicius, Supekar, Menon, & Dougherty, 2009; Vann et al., 2009; van Groen & Wyss, 1990, 1992, 2003). This is particularly true of AThal, where RSC shares dense reciprocal connectivity with a number of different nuclei (Wright, Erichsen, Vann, O'Mara, & Aggleton, 2010; van Groen & Wyss, 2003). These connections are not just structural. The two regions influence one another's processing of space and together help support navigation (Jankowski et al., 2013; Clark, Bassett, Wang, & Taube, 2010), especially when it requires the use of environmental landmarks (Yoder et al., 2011). However, despite the large amount of evidence of mutual interaction between RSC and AThal in rodents, there are far fewer examples of similar communication in humans, only evidence of similar directional coding within both regions (Shine et al., 2016). RSC and anterodorsal parts of the thalamus were previously shown to be more active in good compared with poor navigators when viewing permanent landmarks (Auger et al., 2012). The current study builds upon this finding using a more explicit measure of people's understanding of landmark permanence. This revealed that, rather than simply being coactive, RSC actually drove activity in AThal, and as this directional coupling increased, the better participants had learned which landmarks were permanent.

RSC and AThal both contain neurons that display tuning to the direction an animal's head is facing (Vann et al., 2009; Taube, 1995; Chen et al., 1994). It is therefore interesting that we also observed increased RSC interaction with a third region known to contain head direction cells, the subiculum, albeit at a lower statistical threshold (Taube et al., 1990). Therefore, these three regions, which have consistently been shown to be densely interconnected and functionally related in rodents, were here, in humans, interacting in accordance with the extent of a person's knowledge about permanent landmarks. That said, it could be argued that, given the survey-type paradigm we used here, head direction cells would be unlikely to contribute to performance on this task. Consequently, some other mechanism that is not centered on head direction cells might underpin the permanence response. One way to examine this in the future would be to directly compare permanent landmark representations in small- and large-scale space. If the magnitude of RSC permanence responses is similar, this might suggest that head direction cells are not of prime relevance in this case.

It is also interesting to note that the subiculum is directly implicated along with RSC in the spread of pathology in Alzheimer's dementia (George et al., 2014). This provides further evidence that the disorientation usually present in the early stages of the disease could be a consequence of aberrant processing in RSC, one of the first regions to show pathological changes in this condition (Tu et al., 2015; Pengas et al., 2012; Pengas, Hodges, Watson, & Nestor, 2010). The profound disorientation commonly associated with lesions that involve the RSC (Vann et al., 2009; Maguire, 2001) could also be explained by the failure to identify permanent features, with deleterious effects on environmental representations.

Overall, therefore, we conclude that the coding of permanent landmarks in RSC may drive processing in regions like AThal and subiculum, with possible implications for the efficacy of functions such as spatial navigation.

## Acknowledgments

E. A. M. is funded by a Wellcome Principal Research Fellowship (101759/Z/13/Z) and by a Wellcome Centre Award (203147/Z/16/Z). S. D. A. was funded by UCLH/UCL, who received a proportion of funding from the Department of Health's NIHR Biomedical Research Centres funding scheme. We thank the Imaging Support and Cognitive Interface teams for technical assistance.

Reprint requests should be sent to Eleanor A. Maguire, Wellcome Centre for Human Neuroimaging, Institute of Neurology, University College London, 12 Queen Square, London WC1N 3BG, United Kingdom, or via e-mail: e.maguire@ucl.ac.uk.
